# Perspective On Excellence in Forensic Mental Health Services: What We Can Learn From Oncology and Other Medical Services

**DOI:** 10.3389/fpsyt.2019.00733

**Published:** 2019-10-18

**Authors:** Harry G. Kennedy, Alexander Simpson, Quazi Haque

**Affiliations:** ^1^Department of Psychiatry, Trinity College Dublin, Dublin, Ireland; ^2^National Forensic Mental Health Service, Central Mental Hospital, Dundrum, Dublin, Ireland; ^3^Division for Forensic Psychiatry—University of Toronto Institute of Health Policy, Management and Evaluation, University of Toronto, Toronto, ON, Canada; ^4^Elysium Healthcare, London, United Kingdom; ^5^Division for Forensic Psychiatry—University of Toronto Institute of Health Policy, Management and Evaluation, Dalla Lana School of Public Health—University of Toronto, Toronto, ON, Canada

**Keywords:** excellence, quality, forensic - psychiatric practice, hospital, tiered

## Abstract

We propose that excellence in forensic and other mental health services can be recognized by the abilities necessary to conduct randomized controlled trials (RCTs) and equivalent forms of rigorous quantitative research to continuously improve the outcomes of treatment as usual (TAU). Forensic mental health services (FMHSs) are growing, are high cost, and increasingly provide the main access route to more intensive, organized, and sustained pathways through care and treatment. A patient newly diagnosed with a cancer can expect to be enrolled in RCTs comparing innovations with the current best TAU. The same should be provided for patients newly diagnosed with severe mental illnesses and particularly those detained and at risk of prolonged periods in a secure hospital. We describe FMHSs in four levels 1 to 4, basic to excellent, according to seven domains: values or qualities, clinical organization, consistency, timescale, specialization, routine outcome measures, and research. Excellence is not elitism. Not all centers need to achieve excellence, though all should be of high quality. Services can provide each population with a network of centers with access to one center of excellence. Excellence is the standard needed to drive the virtuous circle of research and development that is necessary for teaching, training, and the pursuit of new knowledge and better outcomes. Substantial advances in treatment of severe mental disorders require a drive at a national and international level to create services that meet these standards of excellence and are focused, active, and productive to drive better functional outcomes for service users.

## Introduction

Forensic mental health services (FMHSs) provide treatment for persons with severe and disabling mental disorders in conditions of therapeutic safety and security. Typically, patients are admitted to forensic services and forensic hospitals from other hospitals, the community, or the criminal justice system because of violent behavior toward others and undergo relatively prolonged, intensive, and restrictive care and treatment (1, 2). By forensic services, we are referring to a therapeutically safe mental health service to a selected population, not only forensic hospitals but also integrated, coordinated systems of care across the interface of criminal justice and mental health services. FMHSs are high cost, low volume, and high risk and must therefore yield high value in health gains. The delivery of clinical services for persons with severe and enduring mental illness is poorly organized, and standards of practice are highly variable. Care can be inconsistent, with variation in standards and in models of care. Best evidence is not routinely implemented. Other parts of the health sector have been more effective at implementing consistent service standards, including treatment as usual (TAU) linked to clinical trials and service research aimed at driving improvement. Quality programs are necessary to ensure that services meet a minimum standard (3–6). Other health services (for example, surgery, oncology, obstetrics) have adopted systems that greatly enhance quality and also continuously improve outcomes for patients through promotion of an academic mission.

This paper describes how excellence can be recognized from the ability to conduct randomized controlled trials (RCTs) (7) and information-driven research paradigms which may be “hypothesis free” (8) or mix quantitative and qualitative methods so as to continuously improve TAU. This is set out with particular reference to FMHS for persons presenting serious risk to others (9), since FMHSs increasingly provide the main access route to more intensive, more organized, and more sustained pathways through care and treatment (10). In FMHS, TAU has been inadequately defined. Scientific evidence is limited for many key practices and interventions (11–14), presenting challenges for the delivery of better outcomes. We will relate this system for describing excellence to the tiers of clinical organization that exist in mental health services generally. FMHS can be structured in a population-based and tiered way to ensure high quality of care through coordinated, robust TAU and continuously improving outcomes through academic leadership of services that promote excellence through research, evaluation, and dissemination of improving clinical standards.

Cancer services provide a model for tiered, population-based specialist care and treatment for serious disorders with some centers of excellence driving the acquisition of new knowledge and better outcomes. A newly diagnosed patient may present to any specialist cancer service and expect evidence-based, systematically implemented, and effective TAU, tested and validated by RCTs, e.g., Giacchetti et al. (15).

With such well-established TAU in cancer services, it is common for newly diagnosed patients to be offered enrolment in international multicenter RCTs comparing TAU with new treatments organized by not-for-profit research cooperatives (16–19).

Like oncology services, FMHSs also treat people with severe illnesses that require consistent, coordinated care pathways and would benefit greatly from this structure of service organization. In this paper, we will describe an organizing framework based on excellence, to advance these ends.

## A Framework to Improve TAU and Achieve Excellence

FMHSs are not as well structured as cancer services when it comes to systematically defined evidence-based treatments, nor for tiered structure and expectations of services. It is notable that there are few high-quality studies of even basic pharmacological interventions for acute psychoses with disturbed behavior (20, 21). It may be argued that psychiatric patients and problems are too complex, and results are too difficult to generalize from RCTs, but these problems can be overcome (22). RCTs are possible for clinically and legally complex non-pharmacological, systemic interventions (23–26) including FMHS (27–31), although the ethical difficulty of producing such studies in a forensic setting is recognized (32–34). This suggests that a new organizing framework is needed.

A structured approach is required to frame service expectations, organization, and values. To achieve this, we propose that clinical quality of forensic psychiatric hospital services should be classified hierarchically. This does not imply a value judgment. Although expressed in terms of a hierarchy, each level includes and builds on the previous levels. Patel et al. (35) suggest that the highest level of “excellence” in relation to clinical services may be taken to mean “outstanding fitness for purpose and surpassing ordinary standards through deliberate practice.” In clinical services, this is true at the level of lead practitioners when related to expertise (36) and at the level of institutional culture (37).

We propose that services can be divided into four broad levels of organization and complexity and can be described by seven domains or qualities (see **Table 1**). Level 1 corresponds to individual practitioners or independent expert clinical groups. Level 2 describes local multidisciplinary teams (MDTs) or groups of services. Level 3 refers to integrated community and hospital services with a broad service mandate, and Level 4 refers to academically led and productive centers of excellence. The seven domains characterizing these levels have been derived from formats or structures for models of care (35) and process mapping (38). The domains are a) values and rights, b) clinical organization, c) consistency, d) timescale, e) specialization, f) routine outcome measures, and g) research and development. We suggest that this approach is relevant to models of care at the systems and population level, and such models of care (35) describe existing structures, processes, and values as a general system (39). These elements of models of care can be distinguished from broader conceptual or professional models such as the bio-psychosocial model (40, 41). A model of care is distinct from such models (42) and may be thought of as elements of a meta-model for systems addressing larger populations and across cultures (43–45).

**Table 1:** Framework for Health Sector Organization to Promote Excellence.

	Level 1	Level 2	Level 3	Level 4
Values/rights	Individualization	Professionalism	Consistency and evidence-based practice treatment as usual (TAU) to address delivery	As 3 plus academic resources and skills
Clinical organization	Independent clinicians/disciplines	MDTs	Hospital governance	As 3 plus national and international networks
Consistency	None	Within team only, some manualized treatment programs	Admission criteria and admission panels, evidence-based leave and tribunal reports	As 3 plus increased measurement, stage of progression, neuropsychological and genetic profiling
Timescale	Day to day	Week to week	Monthly, quarterly, annual	Five-year plans and continuous cycles
Specialization	Patient to patient, qualitative	Small units—gender, diagnostic, security levels	Medium term intensive, longer term slow stream;precision medicine	TAU is defined and disseminated; aspires to personalized medicine
Routine outcome measures	Qualitative	Dynamic only, risk, needs assessment	Functional outcomes linked to evidence-based governance reporting	As 3 plus six monthly ROM
Research	Case studies	Case series	Retrospective and prospective cohort studies	As 3 plus multicenter randomized controlled trials; population-based epidemiology; molecular, imaging, and epidemiological translational research

MDT, multidisciplinary team; TAU, treatment as usual; ROM, routine outcome measurement.

Because complex interventions (46) are required for severe and enduring mental illnesses (SEMIs), Levels 1 and 2 described here may provide services such as primary health care services to persons with SEMI but are unlikely to have a major place in FMHS for severe mental illnesses except when subsumed within Level 3 or 4 services. This integration is itself an enhanced level of integrated and coordinated systems across criminal justice, mental health, and other social institutions and services (47). Prison in-reach and court diversion services provide frontline services typically with systematic screening for severe mental illness (48) and diversion from the criminal justice system (49–52) including youth justice (53) but typically are organized at a regional level and have characteristics of and are integrated with at least tier 3. Impact evaluations of tier 1 forensic services such as street and police station diversion are rare, with benefits emerging when mixed methods participant-action research approaches are adopted in tier 2 or 3 services to modify models of care and systematically evaluate impact and health benefits across a defined population (47, 50, 53–55). The achievement of high quality in the provision of TAU is the primary goal of Level 3 services. The advancement of new knowledge, interventions, and services to improve outcomes is the mission of Level 4 services (see **Table 1**).

## Seven Domains

The characteristics of each domain are now described.

1. Values and Rights
  Services provided at Level 1 value individual practice styles governed by rules of professionalism. At Level 2, services value consistency in professionalism, through a combination of education, specialist training, experience, and patient-centered ethics. This generates expertise, typically regulated by professional registration. At Level 3, welfare rights are emphasized requiring services that have the competencies of Level 2 but are also consistent, accessible, rights-based, and evidence based to meet the complex needs of patients and populations. Such centers are subject to service accreditation by a national regulatory body. At Level 4, services further value research, development, and the dissemination of the best “TAU.” This requires academic resources and skills leading to a cycle from research to development, to teaching and training staff for all levels of service, which forms the basis for further acquisition and dissemination of new knowledge. Academic league tables (56–59) or citation indices (60) may be a guide to achievement in this domain. Mechanisms also exist for crediting cultural appropriateness, community change, and innovation (61–63).
2. Clinical Organization
  At Level 1, each professional discipline acts independently of each other. At Level 2, disciplines organize into multidisciplinary teams with leadership typically by the legally responsible psychiatrist. At Level 3, governance systems operate to ensure continuous improvement in quality, ensuring that a minimum standard is reached by all (3–5). Usually this is formulated in a written model of care (38, 46). This should include a description of recovery pathways through care and across services and organizations, for example, from the criminal justice system to FMHS and on to the community (1, 2), including integrated expert systems of care (47). At Level 4, Level 3 systems are expanded to embrace national and international networks for cooperation (64) as well as organization into academic networks to improve effectiveness and run multicenter research studies.
3. Consistency
  At Level 1, consistency is valued less than individual clinical wisdom. At Level 2, consistency applies only within teams with some consistency through the use of international diagnostic systems and manualized treatment programs. At Level 3, hospital or service-wide governance is applied to essential decision making and processes such as applying admission criteria by admission panels (65–69), to plan care pathways across the service, with evidence-based decision making on matters such as care systems (70–73), leave (74, 75), and reports to mental health review tribunals (76–78). At Level 3, precision medicine can be practiced with diagnosis refined into the staging of progression of illnesses and outcomes (79). At Level 4, precision medicine is likely to be enhanced over time by the investigation of personalized medicine (8) including the development of transdiagnostic systems for diagnosis and treatment based on causal factors such as neuroprogression, neuropsychological, and genetic profiles (80–83).
4. Timescale
  At Level 1, interventions are delivered only on a day-to-day basis. At Level 2, it is possible to organize interventions week to week, corresponding to the pattern of working of an individual multidisciplinary team. At Level 3, it is possible to organize individual treatment plans and care pathways into service-wide programs delivered according to monthly, quarterly, or annual cycles. At Level 4, it is possible to prepare, deliver, and evaluate 5-year plans for service level quality improvement and for translational work from laboratory to clinic (84).
5. Specialization
  At Level 1, specialization is dependent upon how each clinician acts in relation to the uniqueness and autonomy of each individual patient. At Level 2, small specialist units can be developed based on fundamentals such as gender, diagnostic category, or levels of therapeutic structure and support such as levels of therapeutic security (1, 2). At Level 3, it is possible to organize delivery of care and treatment according to stratified care pathways matching needs into acute and sub-acute treatments, medium-term intensive treatments, or longer-term slow-stream treatments (1, 2, 85). This aims to target specific elements responsible for pathology in a given person at a given time within the limits of precision medicine. At Level 4, multimodal TAU can be defined, developed, delivered, and evaluated (86) in research paradigms. These aspire to the investigation of personalized medicine as and when molecular (81, 83), imaging (87, 88), and lifestyle data become available and amenable to individualized use.
6. Routine Outcome Measures
  At Level 1, only qualitative assessment is possible. At Level 2, only dynamic assessment of risks and needs can be assessed over time. At Level 3, functional outcomes can be assessed at regular intervals and linked to evidence-based governance reporting (89). At Level 4, systematic routine outcome measurement becomes normal and is organized as part of service governance and decision making (57). Level 4 services will typically have inter-operable data platforms to allow benchmarking of outcomes on a condition-by-condition basis across a whole cycle of care (89, 90). Routine outcome measures may be organized independently of treating clinicians and blind to interventions at the psychometrics laboratory level, as would be the case in an RCT, or any general medical or surgical hospital.
7. Research
  At Level 1, only individual case studies are possible. At Level 2, limited case series are possible (91, 92). At Level 3, retrospective and prospective observational cohort studies become possible (93–95), developing precision medicine. At Level 4, population-based samples for informatics research (8, 96) and multicenter randomized controlled trials or systematic reviews of treatment trials (97–99) become integrated into systematic treatment along with population-based epidemiological studies as they relate to diagnosis and prediction (100, 101), levels of emergence in personalized approaches (102), and services at a national level (103). At this level, translational research is possible concerning fundamental discovery at the level of molecular, imaging, psychosocial, or epidemiological personalized medicine, from bedside to clinical trials of applications, from translation to policy and health care guidelines, with later assessment of health policy and usage, locally and internationally (84).

## Application to FMHS

FMHSs lend themselves very well to this form of analysis. Like cancer services, they deal with clinical disorders that are potentially life threatening, expensive, and safe if carefully delivered. Services are typically organized in tiers according to the size of the population served. Second-tier services (analogous to Level 3) are provided for populations of approximately 250,000 to 300,000, tertiary services (which should aspire to be Level 4 services) for 3 to 5 million, fourth-tier services for highly specialized sub-populations, and in some large centers, highly specialized services for populations of 20 to 50 million (1, 2, 85).

This population tiering should also be related to quality and excellence, but often, this is not the case. In other areas of clinical practice, it has been shown that a minimum level of experience is required for acceptable outcomes per procedure (104–106), and a minimum volume of new cases is required to maintain both expertise and training functions in many areas of practice (107–111). This results in benefits measured in mortality and side effects even when adjusted for case mix (112, 113). Alternatively, networks of expertise—a different model for delivering centers of excellence—appear to have a positive effect on quality of care if they have adequate resources, credible leadership, efficient management, effective communication, and collaborative, trusting relationships, though the evidence for this is weak (114).

Psychiatric practice should currently be at the level of precision medicine at least in part. Precision medicine (81, 115) is defined as relating to the staging (80–83) and stratification of subgroups of populations for diagnosis, treatment, or prevention, which may result in the targeting of specific elements responsible for pathology in any given person at a particular time (80). This includes the use of diagnostic tests or staging tools for stratification based on the risk of disease or response to treatment (53). These techniques include the structured professional judgment instruments commonly used in forensic mental health such as risk assessment, assessment of functional mental capacity, and triage for stratified therapeutic security (116–119). FMHSs are generally already practicing precision medicine in relation to staging and stratification, though progress is gradual to date in some areas of research (88). Our position is that it is now possible to begin moving toward personalized medicine, as all other areas of practice do so, and that FMHSs are well placed to make this move.

We have cited several studies in which the measurement of complex TAU has been related to personal recovery (78), functional recovery including moves to less secure places and conditional discharge (70, 77) including an RCT (31), and stratification (71, 72), as has been recommended elsewhere (89, 90).

RCTs should be widely used to assess and improve interventions and outcomes in mental disorders and could flourish in the structure proposed here. Interventions for rehabilitation and recovery including violence reduction and prevention in schizophrenia and other mental disorders are multimodal (120). The best evidence for effectiveness in mental health interventions arises from common factors such as therapeutic rapport, working alliance, and motivation (121). Complex interventions are increasingly recognized as amenable to study. Complex interventions are central to an understanding of TAU in randomized controlled trials of psychological treatments in fields such as oncology (122). In FMHS, models of care divide broadly into a standard model of stratified therapeutic security (1, 2) with parallel pathways for special groups and non-stratified units for special subgroups. Comparisons of models of care (24), legal interventions (23, 25), and complex interventions such as integrated care pathway approaches are themselves amenable to randomized controlled trials and systematic study (26).

The EU Joint Programme—Neurodegenerative Disease Research (JPND) (115) provides another example of a system of standards that could be relevant to disabling mental illnesses such as schizophrenia. The large variability in neurodegenerative disorders represents a major impediment to finding optimized approaches to care. Deciphering this variability is therefore necessary. The Horizon 2020 advisory group put forward personalized medicine as a model that uses characterization of individual phenotypes and genotypes (molecular profiling, imaging, and lifestyle data) to tailor the correct individual therapeutic strategy, determining the predisposition to disease, or to deliver timely and targeted prevention (123, 124). The JPND is a trans-national network that is an exemplar of the type of initiative that could substantially improve outcomes for the patients of FMHS.

## Discussion

The most cogent criticisms of FMHSs are not simply debates about the ethics of compulsion but criticisms of effectiveness, and these are based on the absence of RCT studies relevant to the field (14). We have described guiding principles to organize levels of practice within FMHS that will facilitate the development of TAU. All FMHSs could function at what we describe as Level 3, linking to Level 4 FMHSs that are in turn part of national or international networks. A working example can be found in the Scottish FMHSs managed care network (forensic network) established in 2003 (125) and the Expertisecentrum Forensische Psychiatrie in Utrecht, Netherlands (126). We believe there are other informal networks of this type in existence, and organizations such as the International Association of Forensic Mental Health Services encourage such integration of knowledge and service advancement.

The levels described here are not the same as the tiers often described as ways of organizing mental health services according to population size (127, 128). Nor are these levels the same as the tiers of specialization and sophistication for the delivery of more complex interventions. Mental health interventions range from widespread and basic programs for simple or minor problems to more complex care plans and intensive, prolonged interventions for selected patients with disabling or high-risk disorders (128–131). This descriptive system for levels of quality and excellence is compatible with the organization of services into tiers, commencing with basic interventions for minimally disabling disorders affecting a wide population base and increasing to specialized interventions for those with more severe and complex needs. Treatment may reduce levels of dependency, though in the present state of knowledge, evidence that mental health services for severe and disabling mental illnesses lead to improved functional outcomes is limited (132).

Academic center affiliates should deliver “excellent” treatment at Level 4. Setting quality standards and guidelines for TAU is primarily an activity of Level 4 services working in networks which include Level 3 services.

Level 4 FMHS should be peer-reviewed and accredited. This should be both national and international in order to develop the networks needed to foster multicenter RCTs and data-driven research on outcomes. Some national networks for quality and accreditation already exist, such as the quality networks in the UK (3–4, 5, 9) and the Netherlands (6). Up until now, these have focused on raising the minimum standard and fostering consistency. We believe such networks can better drive excellence by moving from primarily judging compliance with standards and guidelines to also accrediting quality improvement by services using research-informed outcome measurement and management (9, 133). International networks such as the International Association of Forensic Mental Health Services, European Psychiatric Association, and World Psychiatric Association are also focused on these goals. The ambition should be to emulate what has been achieved by cancer research networks (16–19) and networks in other areas (134).

Characteristics described for Level 4 excellence would be regarded as standard in most oncology services. There are very few such excellent services available for people with disabling mental illnesses and disorders. There is no reason why this should not now be possible. Much of the infrastructure for Level 4 services already exists in FMHS organized at regional and national levels. We believe that in general, most such services are operating or can operate at Level 3, but often with inadequately defined TAU and models of care. It is now possible to ensure that TAU is consistent, evidence based, and improving through international cooperation, when also adapted thoughtfully to local population needs.

We are concerned with systemic ways of improving measurable “real-world” outcomes such as survival, personal recovery and hope, symptomatic recovery, functional recovery, recovery of autonomy and responsibility (forensic recovery), and reduced length of stay. We have presented a detailed analysis of research literature on how these fundamentals have improved markedly in other areas of clinical practice, notably oncology but also obstetrics and surgery. We have drawn attention to the disappointing lack of progress in these domains in services for the most severely mentally ill. We have extensively cited RCTs of psychological and psychosocial interventions in forensic psychiatry (27–34), while noting how sparse these are (12–14). We believe that the addition of mixed methods to study “soft” or “subjective” outcomes is also important (78, 89), and these are useful insofar as they contribute to improving such outcomes as survival, earlier discharge from secure places (77), reduced risk of violence (86, 93, 94, 96, 97, 99, 120) and suicide (103), or improved function (86, 89).

We have reviewed the viability of RCTs for complex interventions in psychiatry (7, 22). Manualization is no longer regarded as the most effective aspect of delivering larger effect sizes with fidelity to the treatment modality (121). Real-world data analytic studies may be a better approach to identifying what may be effective (8, 87, 101, 102, 124). RCTs in psychiatry will require the capacity to quantify complex TAU (32), and this will depend on aggregation of measures (77, 78, 86, 116–119). In detecting treatment effects, it is important to use measures that are calibrated in units of meaningful change rather than arbitrary units, an approach that is uniquely possible in forensic services (119).

It is notable that some of the best models or meta-models for psychiatric care omit references to FMHS and excellence (135, 136). This can be contrasted with recent recognition of the interconnectedness of general adult and forensic psychiatric services and pathways (137). We propose that advances in the treatment of the more severe mental disorders require a drive at a national and international level to create forensic psychiatry centers of excellence. Services that are focused, active, productive, and excellent are required in order to drive better outcomes for service users.

## Ethics Statement

The authors assert that all procedures contributing to this work comply with the ethical standards of the relevant national and institutional committees on human experimentation and with the Helsinki Declaration of 1975, as revised in 2008, and the authors assert that all procedures contributing to this work comply with the ethical standards of the relevant national and institutional guides on the care and use of laboratory animals.

## Author Contributions

HK, AS, and QH contributed conception and design of the study. HK wrote the first draft of the manuscript. All authors contributed to manuscript revision, read and approved the submitted version.

## Conflict of Interest

The authors declare that the research was conducted in the absence of any commercial or financial relationships that could be construed as a potential conflict of interest.
